# Supplementary material for: The causal relationship between the human gut microbiota and pyogenic arthritis: a Mendelian randomization study
Source: Front Cell Infect Microbiol. 2024 Nov 26;14:1452480. doi: 10.3389/fcimb.2024.1452480 (PMC11629706; doi:10.3389/fcimb.2024.1452480)
Supplement: Supplementary file 1 [file DataSheet1.zip › mendelian test/ebi-a-GCST90027644.csv_forest.pdf]

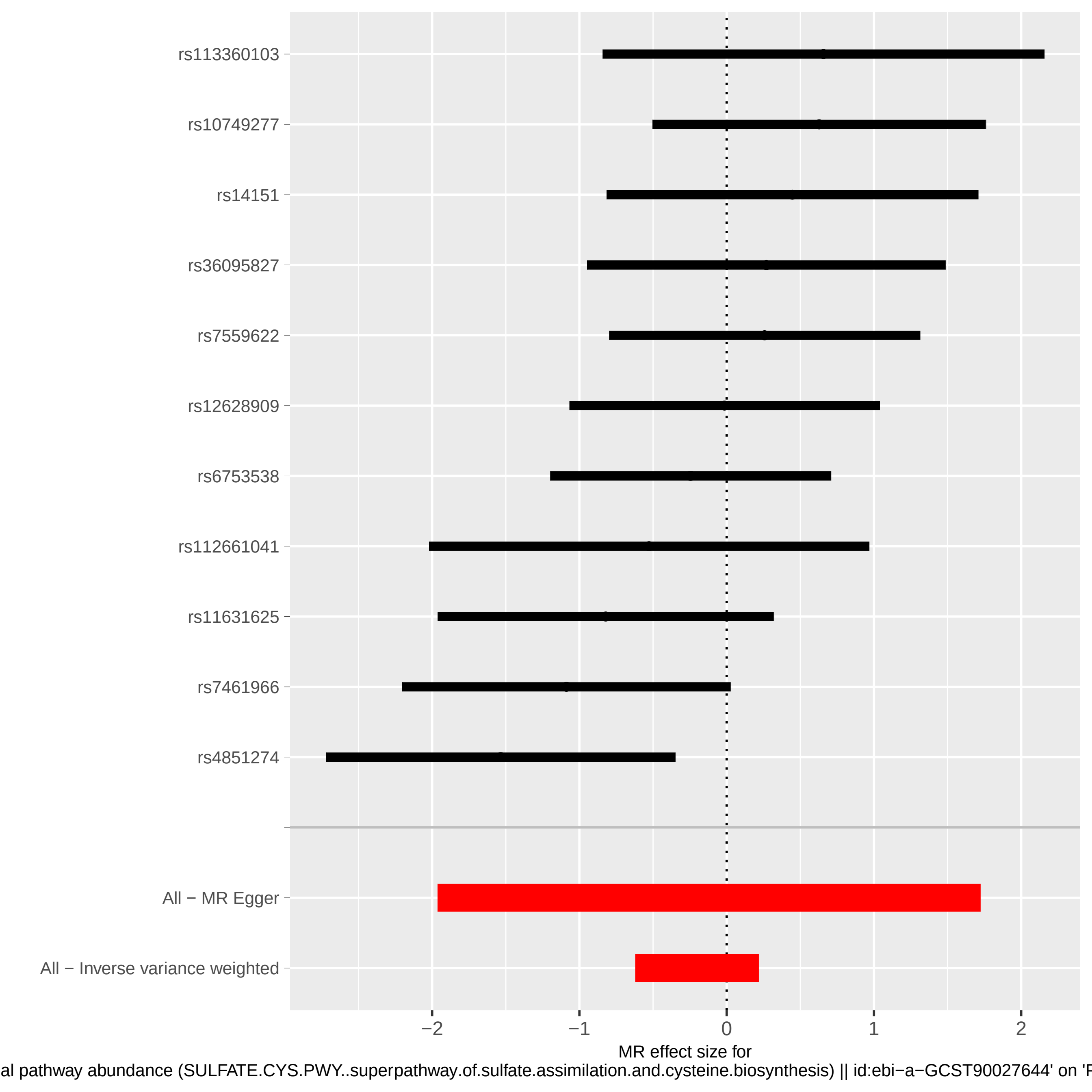
rs113360103
rs10749277
rs14151
rs36095827
rs7559622
rs12628909
rs6753538
rs112661041
rs11631625
rs7461966
rs4851274
All − MR Egger
All − Inverse variance weighted
−2
−1
0
1
2
MR effect size for
al pathway abundance (SULFATE.CYS.PWY..superpathway.of.sulfate.assimilation.and.cysteine.biosynthesis) || id:ebi−a−GCST90027644' on 'F
